# Deep analysis of skin molecular heterogeneities and their significance on the precise treatment of patients with psoriasis

**DOI:** 10.3389/fimmu.2024.1326502

**Published:** 2024-03-01

**Authors:** Shengxiao Zhang, Minjing Chang, Leilei Zheng, Can Wang, Rong Zhao, Shan Song, Jiawei Hao, Lecong Zhang, Caihong Wang, Xiaofeng Li

**Affiliations:** ^1^ Department of Rheumatology, Second Hospital of Shanxi Medical University, Taiyuan, Shanxi, China; ^2^ Ministry of Education, Key Laboratory of Cellular Physiology at Shanxi Medical University, Taiyuan, Shanxi, China; ^3^ Shanxi Key Laboratory of Big Data for Clinical Decision, Shanxi Medical University, Taiyuan, Shanxi, China

**Keywords:** gene expression profiling, machine learning, psoriasis, stratification, unsupervised clustering

## Abstract

**Background:**

Psoriasis is a highly heterogeneous autoinflammatory disease. At present, heterogeneity in disease has not been adequately translated into concrete treatment options. Our aim was to develop and verify a new stratification scheme that identifies the heterogeneity of psoriasis by the integration of large-scale transcriptomic profiles, thereby identifying patient subtypes and providing personalized treatment options whenever possible.

**Methods:**

We performed functional enrichment and network analysis of upregulated differentially expressed genes using microarray datasets of lesional and non-lesional skin samples from 250 psoriatic patients. Unsupervised clustering methods were used to identify the skin subtypes. Finally, an Xgboost classifier was utilized to predict the effects of methotrexate and commonly prescribed biologics on skin subtypes.

**Results:**

Based on the 163 upregulated differentially expressed genes, psoriasis patients were categorized into three subtypes (subtypes A–C). Immune cells and proinflammatory-related pathways were markedly activated in subtype A, named immune activation. Contrastingly, subtype C, named stroma proliferation, was enriched in integrated stroma cells and tissue proliferation-related signaling pathways. Subtype B was modestly activated in all the signaling pathways. Notably, subtypes A and B presented good responses to methotrexate and interleukin-12/23 inhibitors (ustekinumab) but inadequate responses to tumor necrosis factor-α inhibitors and interleukin-17A receptor inhibitors. Contrastly, subtype C exhibited excellent responses to tumor necrosis factor-α inhibitors (etanercept) and interleukin-17A receptor inhibitors (brodalumab) but not methotrexate and interleukin-12/23 inhibitors.

**Conclusions:**

Psoriasis patients can be assorted into three subtypes with different molecular and cellular characteristics based on the heterogeneity of the skin's immune cells and the stroma, determining the clinical responses of conventional therapies.

## 1 Introduction

Psoriasis is a common chronic auto-inflammatory skin disease characterized by obviously erythematous and scaly skin lesions accompanied by prominent skin and joint manifestations (1, 2). Chronic plaque or psoriasis vulgaris is the most common form of psoriasis, where lesions arise from hyperproliferation and disrupted differentiation of epidermal keratinocytes provoked by innate and adaptive immune system dysfunction (3, 4). The American Academy of Dermatology proposes biologics as a first-line treatment option for moderate to severe plaque psoriasis owing to their superior efficacy and acceptable safety profile compared to other treatment options. Specifically, the most commonly prescribed biologics are inhibitors to tumor necrosis factor-α (TNF-α; including etanercept and adalimumab) and cytokine-targeting treatments such as the p40 subunit of interleukin (IL)-12/23 (ustekinumab) and IL-17 (brodalumab) (5). The systemic anti-inflammatory response caused by most of these biologics in treating psoriasis decreases the severity of comorbidities. Nevertheless, a considerable number of patients with psoriasis still present an inadequate response to these therapies (6–8), which may result from the heterogeneous pathophysiological background of psoriasis.

Gene expression profiling of psoriasis skin tissues has been used to provide pathophysiological insights for explaining the variations in treatment outcomes. Ainali et al. have revealed two distinct molecular subgroups from a single chronic plaque psoriasis skin transcriptomic profile and noted that one of these subgroups enriched in the transforming growth factor-β and ErbB signaling pathways might be more amenable to biologics therapies (9). Wang et al. have delineated two distinct immune phenotypes and constructed a psoriatic microenvironment score from a meta-analysis of psoriasis skin transcriptomic profiles (10). There is a correlation between improved clinical outcomes and overexpression of genes responsible for keratinocyte differentiation in the nonlesional phenotype. Some characteristics are common among the various subtypes, but their conclusions are inconsistent. Consequently, gaining more profound and comprehensive mechanistic insights into divergent and shared features of the psoriasis skin subtypes is necessary for determining the pathobiological approaches for treatment-resistant patients with psoriasis.

In this study, we aimed to elucidate the cellular, molecular, and clinical features of three newly identified psoriasis skin subtypes using an unsupervised machine learning method performing a comprehensive meta-analysis of publicly available microarray datasets published thus far. Finally, we sought to apply a machine-learning model to identify the psoriasis skin subtypes for evaluating the therapeutic efficacy of biologics.

## 2 Methods

### 2.1 Systematic search, data selection, and preprocessing

**Figure 1:**
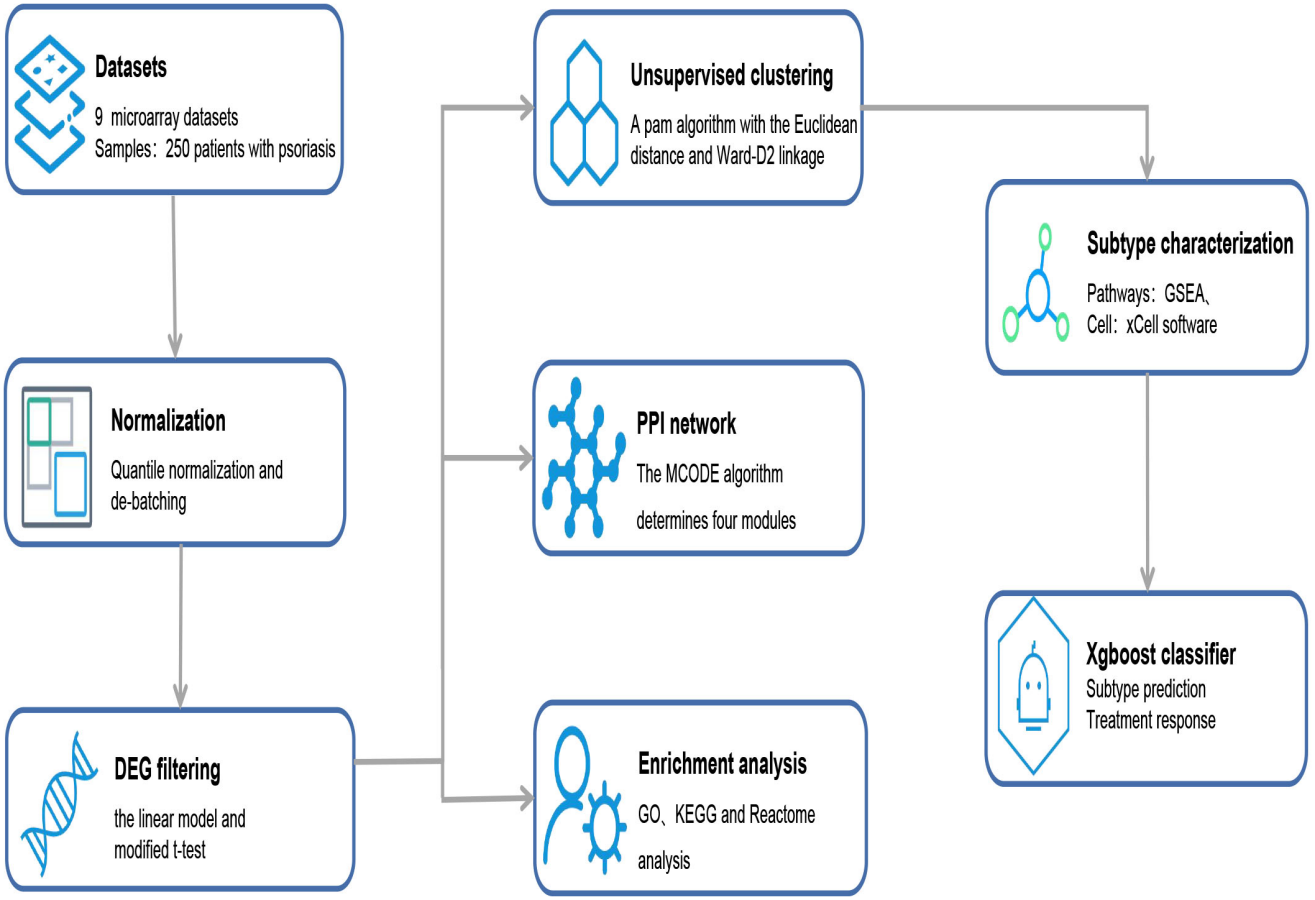
Overview of data processing steps. From the public databases, nine microarray datasets containing 250 patients with psoriasis were selected. According to the established methodologies, DEGs were filtered, and enrichment analysis, PPI network analysis, and supervised clustering were performed. Finally, the Xgboost classifier was constructed to predict the responses of stratified subtypes to commonly used biologics. DEGs, differentially expressed genes; GO, Gene Ontology; GSEA, Gene-set enrichment analysis; KEGG, Kyoto Encyclopedia of Genes and Genomes; MCODE, Molecular Complex Detection; PPI, protein–protein interaction.

The study design is outlined in **Figure 1**. We retrieved the microarray gene expression datasets of psoriasis skin datasets from the Gene Expression Omnibus database. Nine microarray datasets (GSE30999 (11), GSE13355 (12), GSE14905 (13), GSE41662 (14), GSE53552 (15), GSE34248 (14), GSE67853 (16), GSE47751 (17), and GSE50790 (18) of eligible psoriasis skin tissues were collected for this study (**Supplementary Table 1**). The independent cohorts for treatment include 66, 85, 73 and 15 psoriasis lesions that were treated with etanercept (14, 19), ustekinumab (19, 20), brodalumab (20), and methotrexate (21), respectively, to which therapeutic responses were measured using the Psoriasis Area and Severity Index (PASI) score endpoint after the initiation of therapy. Here, patients were considered responders when there was an improvement of at least 75% on the PASI score from baseline and were considered non-responders otherwise.

Using the affy R package, microarray raw data from Affymetrix was processed by employing the robust multi-array average algorithm for background correction, quantile normalization, and probe-set summarization. Normalized matrix files are downloaded directly from Illumina into raw microarray data.

Residual technical batch effects from different data sets were corrected using the ‘ComBat’ function in the sva R package to reduce the systematic, dataset-specific bias (22). Quality assurance and distribution bias were assessed using the principal component analysis (PCA) of the same datasets before and after normalization and batch correction (**Supplementary Image 1**).

### 2.2 Filtering of differentially expressed genes and functional comments

The limma R package performed gene identification by differential expression analysis of microarray data from lesional and non-lesional psoriasis skin tissues using a linear model and modified t-test (23). To control for the proportion of false positive findings, we adjusted P-values using the Benjamini-Hohberg correction (24), and adjusted P-values <0.05 and absolute fold change values ≥1.5 were considered statistically significant. Then, the functional enrichment analysis of the upregulated DEGs lists in psoriatic lesions skin was performed via the Metascape online website. Adjusted p <0.05 was deemed to be significant for enriched functional pathways (25).

### 2.3 Construction of protein-protein interaction network and identification of the important modules

To visualize the interconnectedness of DEGs in the psoriasis skin samples, we constructed a protein-protein interaction (PPI) network based on the STRING and BioGrid databases and Cytoscape software (26, 27). The network is described by proteins (i.e., nodes) and their relationships (physical or functional interactions) (i.e., edges). Four significant modules were identified during the analysis using the Molecular Complex Detection (MCODE) algorithm (28). The biological functions of important module genes were identified by enrichment analysis. Based on the P value, the three highest-scoring terms were retained to describe the function of the corresponding module.

### 2.4 Gene-set enrichment analysis

Gene-set enrichment analysis (GSEA) ranked the list of upregulated DEGs in psoriasis-lesioned skin using a predefined gene set database to identify potential biologically critical pathways related to psoriasis (29, 30). Information on gene sets for signaling pathways or biological processes was obtained from the Kyoto Encyclopedia of Genes and Genomes (KEGG) and Reactome databases. Terms with a false discovery rate below 0.25 were considered significant.

### 2.5 Unsupervised clustering for psoriasis skin gene expression profiles

To classify patients with psoriasis into subtypes based on the skin transcriptomic signatures, we performed hierarchical agglomerative clustering using the ConsensuClusterPlus R package (31). The procedure was repeated for 1000 iterations to ensure the stability of the subtype stratification. The clustering approach adopted a Partitioning Around Medoids algorithm with the Euclidean distance and Ward-D2 linkage, and the optimal and stable number of clusters was selected by the cumulative distribution function (CDF). We then used the PCA to confirm the results of unsupervised clustering. DEGs were differentially expressed in the stroma- and immune-enriched subtypes compared to those differentially expressed in the other subtypes. An adjusted P-value <0.05 and absolute fold-change ≥1.5 were used to identify enriched pathways in the Gene Ontology Biological Process (GO-BP) and Reactome databases.

### 2.6 Inference of cell types and pathways activated in psoriasis skin subtypes

To explore the specific biological pathways associated with the subtypes, we used single-sample GSEA to condense information from gene expression profiles into pathway or marker gene sets (32). Here, the enrichment score is defined as the absolute enrichment of the gene set in each sample of a given dataset. Psoriasis skin-related pathways were collated from the published literature and GSEA results, with gene sets from KEGG and response group databases. We used the “xCell” algorithm to calculate the enrichment scores for immune and stroma cell-type signatures for elucidating the cellular composition of the three subtypes (33). Enrichment scores representing given cell types and pathway activities were compared among the three subgroups using the Wilcoxon and Kruskal-Wallis test.

### 2.7 Construction of multi-classification model for psoriasis skin subtype prediction

The Xgboost [extreme gradient boosting] (version 1.5.0.2) is a machine learning method that assembles weak prediction models to build more robust prediction models. We made a prediction model based on 163 gene features using the Xgboost-tree method with softmax objective function. The predictive power of the system was then measured using the average area under the receiver operating characteristic curve (AUC). For training the classifier, 250 psoriasis skin samples were divided into training (n=176) and testing (n=74) sets in 70% and 30% proportions, respectively, which used the caret R package. We controlled overfitting in the model using 10-fold cross-validation and used the fitted model to select subtypes for the new samples. Finally, we applied the 163-gene classifier for categorizing the samples into subtypes.

### 2.8 Statistical analysis

All statistical analyses were performed using the R software (version 4.0.3). We used the Kruskal–Wallis test to compare differences among two or more samples and the Wilcoxon test to compare two samples. The chi-square or Fisher’s test was applied to analyze the relationship between the psoriasis skin subtypes and clinical measures. Statistical significance was set at P<0.05 (two-tailed test).

## 3 Results

### 3.1 DEGs, PPI network, and enriched signaling pathways

**Figure 2:**
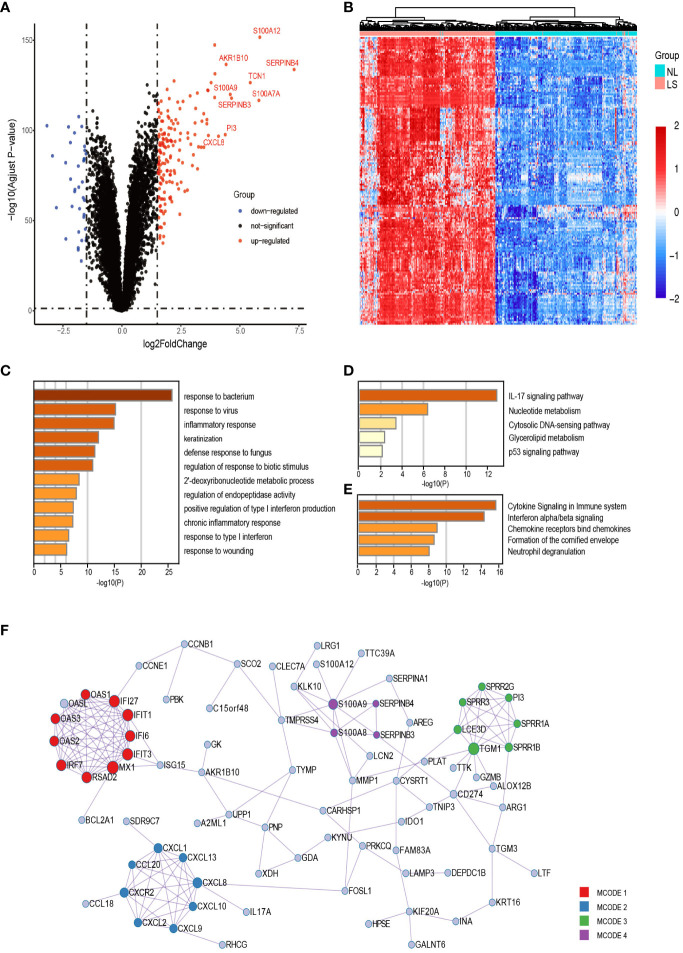
Upregulated DEGs analysis between lesional and nonlesional skin samples of patients with psoriasis. **(A, B)** Volcano plot and heatmap of all the DEGs. **(C)** Functional enrichment analysis of upregulated DEGs. The top 20 most significant biological processes in the GO-BP database. **(D, E)** Pathway enrichment analysis of upregulated DEGs. Top 5 most considerable signaling pathways in the KEGG and Reactome databases. **(F)** Protein–protein network of upregulated DEGs. The nodes and edges of the network represent genes and the functional or physical relationships between them, respectively. Four modules were found to be significant using the MCODE algorithm. GO-BP, Gene Ontology Biological Process.

On comparing the gene expression profiles of lesional and non-lesional skin samples from 250 patients with psoriasis, 163 upregulated DEGs were obtained (**Figures 2A, B**). Gene ontology [GO] annotation demonstrated that these upregulated DEGs in lesional skin were appreciably enriched in biological processes(BP) such as keratinization; inflammatory response; response to viruses, bacteria, and fungi; and type I interferon (IFN) production (**Figure 2C**). The KEGG and Reactome analysis indicated that the upregulated DEGs primarily enriched the IL-17 signaling, p53 signaling, neutrophil degranulation, and IFN-α/β signaling pathways (**Figures 2D, E**).

In total, 188 interactions among 82 DEGs were identified in the PPI networks constructed using these DEGs, and 81 genes were isolated without any direct relation to each other. The MCODE analysis identified 29 hub genes and clustered them into four highly correlated protein clusters (**Figure 2F**). Enrichment analysis was performed separately for each module, and a functional descriptor was chosen for each module based on the three terms that scored best based on the P-value. In line with our expectations, combining the result of GSEA, IFN-related (IFN-α/β/γ signaling and response to viruses) genes, neutrophil-related (neutrophil degranulation and response to bacteria and fungi) genes, toll-like receptor (TLR), NOD-like receptor (NLR), RIG-I-like receptor (RLR) signaling pathways, and keratinization were significantly enriched in lesional skin, adequately explaining the molecular mechanism of psoriasis skin.

### 3.2 Identification of skin gene expression-driven subtypes

**Figure 3:**
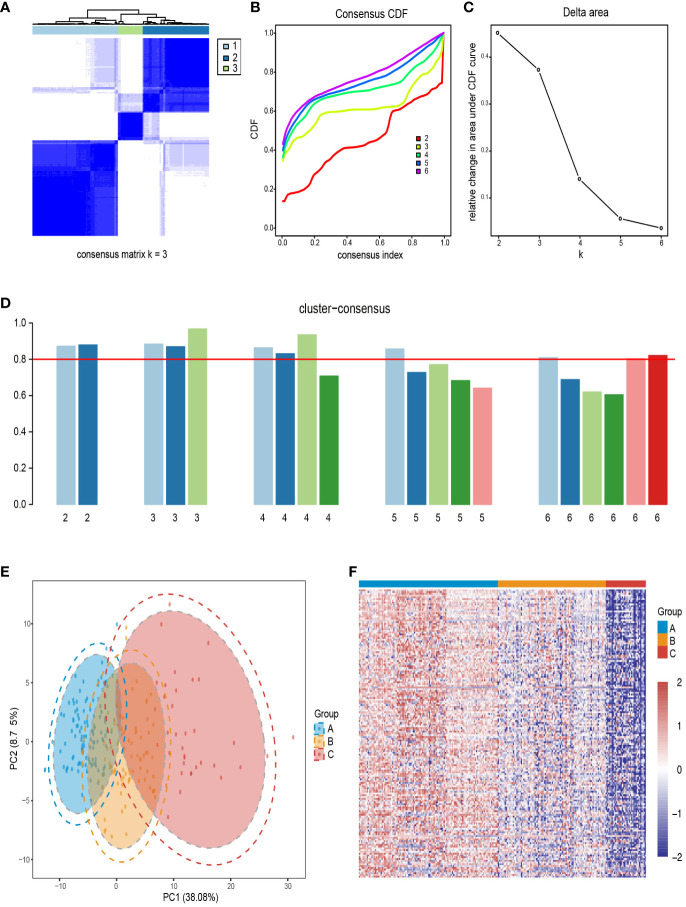
Identification of psoriasis skin subtypes based on upregulated DEGs. **(A)** A recorded consensus matrix at k=3 for the psoriasis skin compendium. The values of the consistency matrix from white to dark blue are from 0 to 1. **(B)** Consensus clustering for the CDF for k=2–6. **(C)** Relative change in area under CDF curve for k=2–6. **(D)** The cluster consistency score for each subgroup of k=2–6, and the red horizontal line indicates a cluster consistency score of 0.8. **(E)** PCA for the DEG expression profiles shows the stability and reliability of the classification. **(F)** A heatmap of 250 patients with psoriasis with a red gradient illustrating the gene expression distribution for three psoriasis skin subtypes. In each column, patients are grouped based on cluster assignment. Red represents overexpression, while blue represents under-expression. CDF, cumulative distribution function; PCA, Principal components analysis.

Using the “ConsensusClusterPlus” R package of 1,000 iterations, we evaluated the number of clusters from k = 2 to k = 6. The CDF value and the delta area were used to measure the robustness of clustering results. The results show that when K=3, the consensus matrix (**Figure 3A**), consensus CDF plot (**Figure 3B**), and delta area plot (**Figure 3C**) all showed stable results, and the clustering consistency scores of each subgroup exceeded 0.8 (**Figure 3D**). Psoriasis skin subtype segregation patterns were revealed using the PCA (**Figure 3E**). The heatmap plot showed upregulated DEG in the three subtypes, showing the gene-level variability of the three subtypes (**Figure 3F**).

**Figure 4:**
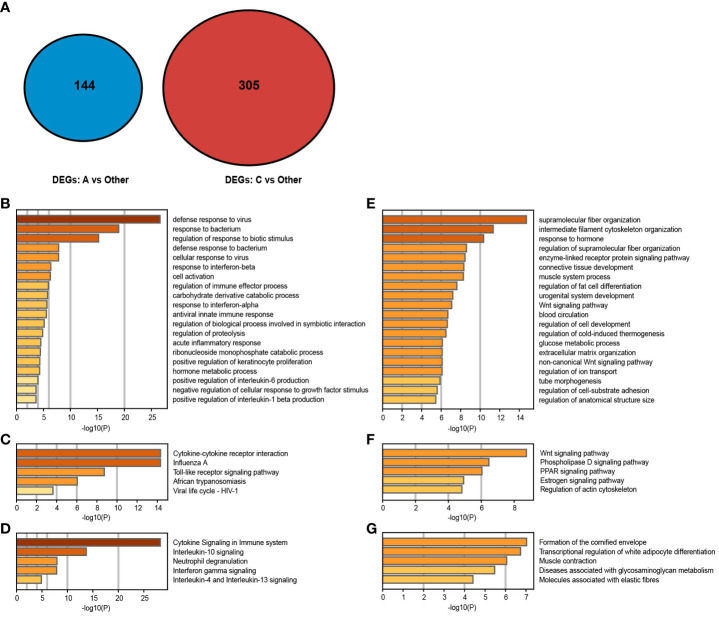
Gene expression patterns of psoriasis skin subtypes. **(A, B)** Molecular pattern distribution of subtypes A and C concerning different biological processes. The top 20 most significant biological processes in the GO-BP database. **(C–F)** Molecular pattern distribution of subtypes **(A, C)** concerning different pathways. Top 5 most considerable signaling pathways in the KEGG and Reactome databases. **(G)** A Venn diagram shows upregulated DEGs in subtypes **(A, C)**.

We compared each cluster with the others (such as subtype A vs subtype B and subtype C) to determine the specific upregulation of DEG signatures in each psoriasis skin subtype and to evaluate the molecular signatures and biological processes of the corresponding subtype. Here, 144 DEGs were significantly upregulated in subtype A, only 1 in subtype B, and 305 in subtype C using the same filtering threshold (**Figure 4A**). Thereafter, the Metascape was used to enrich the most obviously dysregulated biological processes and signaling pathways of each subtype from the Gene Ontology Biological Process (GO-BP), KEGG, and Reactome databases. To be specific, there was a significant activation of canonical immune pathways in subtype A, such as neutrophil-related pathways (including neutrophil degranulation and response to bacteria or fungi) and IFN-related pathways (including response to IFN-α/β/γ and response to viruses) (**Figures 4B–D**). There was an enrichment of stroma proliferating pathways in subtype C, including cornified envelope formation, peroxisome proliferator-activated receptor (PPAR) signaling pathway, and Wnt signaling pathway (**Figures 4E–G**). Additionally, the mixed subtype (subtype B) shared features with both the immune-activating and stroma-proliferating subtypes, whereas upregulated DEGs of these subtypes exhibited no overlap. As it was a blend of the two subtypes, very few genes were unique to the mixed subtype.

### 3.3 Molecular and cellular characterization of the three skin subtypes

**Figure 5:**
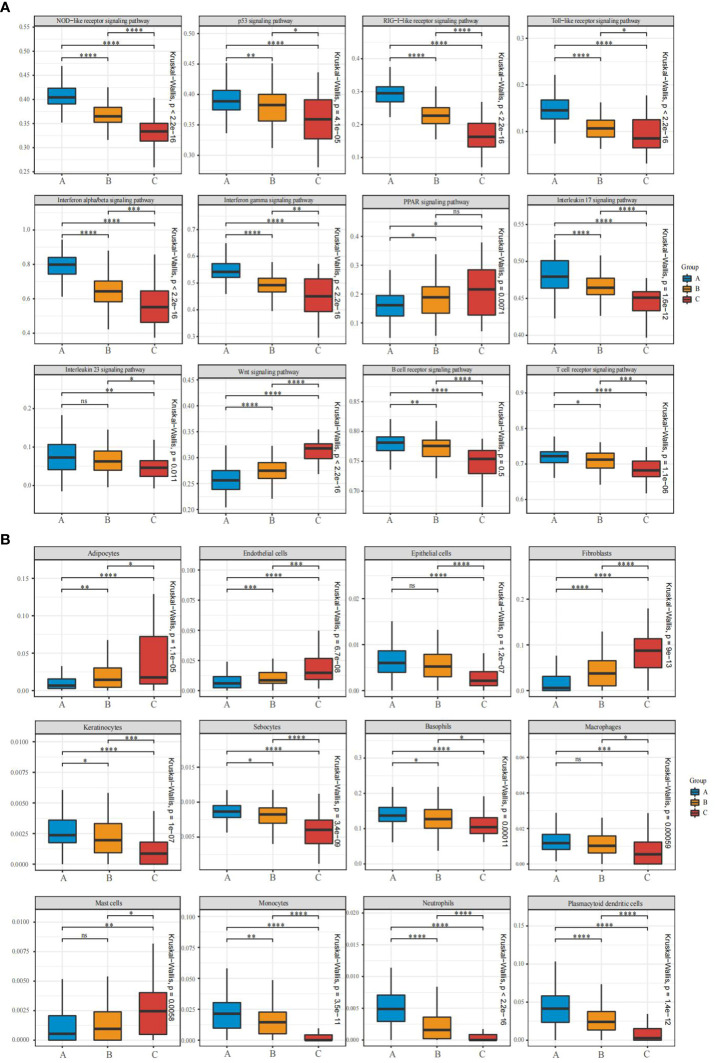
Pathway and cell subset-driven characterization of psoriasis skin subtypes. **(A, B)** Box plots for enrichment scores of pathways and xCell-inferred cell subsets for each psoriasis skin subtype. Differences across the three subtypes were analyzed using the Wilcoxon and Kruskal-Wallis test. ns, not significant; *P<0.05; **P<0.01; ***P<0.001; **** P<0.0001.

There were three clustered subtypes: A (n=121), B (n=94), and C (n=35). To validate whether immune and stromal characteristics differ among these subtypes, we compared enrichment scores of important psoriasis-associated pathways and cell subsets. Twelve psoriasis-associated pathways or processes were curated from the literature and KEGG and Reactome databases. There were significant differences among these subtypes in enrichment scores for these signaling pathways associated with psoriasis. In subtype A, NLR, TLR, RLR, p53, IFN-α/β, IFN-γ, IL-17, IL-23, T-cell receptor, and B-cell receptor signaling were strongly activated, indicating immune activation. Subtype C was characterized by stroma proliferation and enrichment in the Wnt and PPAR signaling pathways, whereas all signaling pathways are moderately activated in subtype B (**Figure 5A**).

The xCell software was used to identify cell types leading to gene expression discrepancy among subtypes, and a machine-learning framework was built to estimate cell type enrichment. All three subgroups showed differential activation of immune and stromal cells, consistent with these expression patterns. Immune cells [including neutrophils, basophils, macrophages, plasmacytoid dendritic cells (pDCs), and monocytes] and stroma cells (including epithelial cells, keratinocytes, and sebocytes) were more activated in subtype A than in subtypes B and C, whereas mast cell activation declined sharply. Moreover, fibroblasts, adipocytes, and endothelial cells were significantly infiltrated in subtype C (**Figure 5B**). An analysis of functional pathways in subtypes of psoriasis confirmed these results.

### 3.4 Prediction of the treatment responses of the subgroups

An Xgboost machine learning algorithm developed a 163-gene classifier to verify the psoriasis subtyping scheme. After training the classifier with 176 psoriasis samples, we applied it to the testing set containing 74 psoriasis samples to confirm its practicality. We observed that the AUC of the training set is 0.932, which proves that the model effectively classifies it. Furthermore, the classifier demonstrated robustness by achieving an average classification performance of 0.905 in the testing set. As a consequence, the classifier was highly effective and applicable in assessing psoriasis skin subtypes.

**Figure 6:**
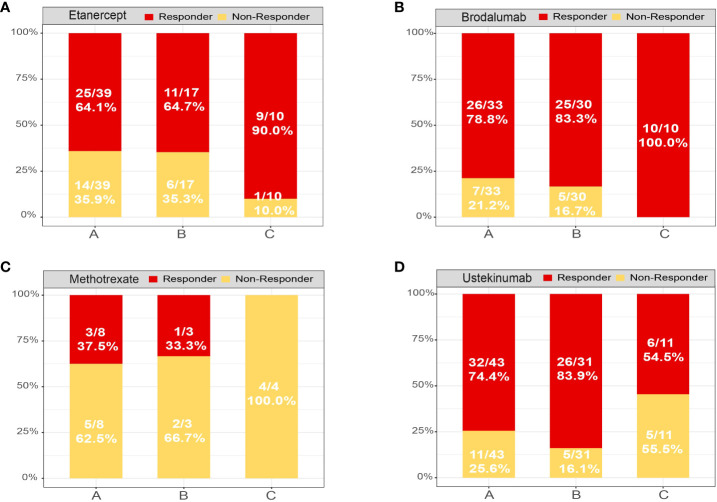
Treatment response of each psoriasis skin subtype. Distribution of psoriasis skin subtypes based on the treatment response of patients treated with **(A)** etanercept, **(B)** brodalumab, **(C)** methotrexate and **(D)** ustekinumab. Response: responded to the biologics; non-response: did not respond to the biologics.

To determine whether the therapeutic response to etanercept, brodalumab, methotrexate (MTX) and ustekinumab were associated with subtypes of psoriasis, we classified patients at baseline using a fitted Xgboost classifier in four datasets. In the etanercept and brodalumab treated groups, the proportion of good responders was significantly higher in subtype C [9/10 (90.0%) and 10/10 (100.0%)] than in subtypes A [25/39 (64.1%) and 26/33 (78.8%)] and B [11/17 (64.7%) and 25/30 (83.3%); **Figures 6A, B**]. In contrast, an opposite trend was observed in the MTX-treated group; the proportion of good responders was higher in subtypes A [3/8 (37.5%)] and B [1/3 (33.3%)] than in subtype C [0/4 (0.0%); **Figure 6C**]. An excellent response to ustekinumab was more frequently observed in subtypes A [32/43 (74.4%)] and B [26/31 (83.9%)] than in subtype C [6/11 (54.5%; **Figure 6D**]. In spite of this, the differences were not statistically significant, likely due to the limited sample size. In our analysis, subtypes A and B exhibited good responses to MTX and IL-12/23 inhibitors (i.e., ustekinumab) and inadequate responses to TNF-α inhibitors (etanercept) and IL-17A receptor (IL-17RA) inhibitors (i.e., brodalumab) compared with subtype C. Collectively, psoriasis molecular subtyping could have an impact on the benefits of drug treatment. Future clinical studies should integrate this information.

## 4 Discussion

Studying the molecular features of skin stratification in psoriasis has improved our understanding of its biological complexity and clinical heterogeneity and promoted research on psoriasis. Although previous studies have emphasized that stromal-cell-rich subtypes may respond well to existing biological therapies (9, 10), adequate information on approaching treatment-resistant patients with psoriasis remains lacking. Here, we classified the psoriasis skin tissues based on their molecular signatures using unsupervised cluster analysis (31). We characterized the different features of the three clustered subgroups in cellular components and biological processes and explained the results from therapeutic perspectives. In detail, subtype A (named immune activation subtype) had a transcriptomic signature of immune cells and proinflammatory activation-related pathways. In contrast, subtype C (designated as the stroma proliferation subtype) exhibited more enriched transcripts in stroma cells (such as fibroblasts and endothelial cells) and tissue proliferative-related pathways, while the features of subtype B (named as mixed subtype) fell somewhere in between. Notably, the therapeutic responses were different among these three subgroups.

Currently, 20-30% of patients do not respond to FDA-approved treatments for psoriasis (34). Therefore, correct disease stratification and active exploration of the response of Psoriasis patients to different treatments are the first steps in the precision treatment of Psoriasis. In the past few years, Wang et al. analyzed genome-wide mRNA expression in psoriatic skin biopsies, discovered different immune cell infiltration patterns that distinguish psoriatic lesions from healthy skin, and successfully classified psoriasis into two different immune phenotypes. Among them, the nonlesional phenotype was characterized by overexpression of genes involved in keratinocyte differentiation and referred to as associated with better treatment response of biologics (10). This conclusion is highly consistent with our findings but has not provided an adequate explanation and address on how to approach it. Our results indicate that subtype C was strongly enriched with proliferative tissue pathways, including PPAR and Wnt signaling, and cellular components, such as fibroblasts and endothelial cells, but not for immune cells and proinflammatory signaling pathways. This finding indicates that immune cell activation was not necessarily involved in skin destruction and may provide a reasonable explanation for the conflict between the low level of inflammatory markers and continuous disease progression on the part of patients with psoriasis. Patients with subtype C presented good responses to TNF-α inhibitors (such as etanercept) and IL-17RA inhibitors (brodalumab). Previous studies have shown that a small amount of TNF-α and IL-17 can act on fibroblasts and endothelial cells to stimulate and produce numerous proinflammatory and proliferative cytokines, such as IL-6, IL-8, and CXCL-1, which cause excessive proliferation of psoriatic skin epidermis (35). Notably, Zaba et al. found that the efficacy of TNF-α inhibitors was associated with IL-17RA inhibition, as rapid downregulation of IL-17A pathway-related gene expression was observed in patients who responded to etanercept but not in the non-responders (36). These results suggest that the current therapeutic strategies targeting TNF-α and IL-17RA could almost wholly inhibit the skin pathology in the good responders. Interestingly, brodalumab’s effects on fibroblasts and endothelial cells have been demonstrated in systemic sclerosis (37). Brodalumab reduces fibroblast proliferation and collagen production by maintaining the regulatory T (Treg) cells/T helper (Th) 17 balance (38), resulting in reduced dermal thickness and improvement in modified Rodnan skin score. In addition, Takemichi Fukasawa et al. found that after six months of guselkumab (IL-23 inhibitor) being used to treat patients with psoriasis vulgaris complicated by systemic sclerosis, both Th2 and Th17 cells showed a decline, and the severity of the disease was also significantly improved (39). These findings further confirm that the IL-23/IL-17 axis is an essential pathway for targeted therapy of inflammatory skin diseases.

Activation of the inflammatory profile of subtypes A may account for the positive results of current psoriasis treatment targeting the upstream immune-inflammatory response. Patients with subtypes A were relatively sensitive to disease-modifying anti-rheumatic drugs (DMARDs) (i.e., MTX) and IL-12/23 inhibitors (i.e., ustekinumab). The anti-microbial peptide LL-37 is primarily secreted by keratinocytes and immune cells in the early stages of psoriasis development through direct activation of pDCs and myeloid dendritic cells to secrete IL-12 and IL-23 (40–42). Moreover, IL-12 and IL-23 stimulated TNF-α and IL-17 secretion in Th1 and Th17 cells (43, 44), resulting in a strong skin inflammatory reaction. Ustekinumab was designed to prevent the proliferation of the series of immune cells and the secretion of various proinflammatory cytokines by blocking IL-12/23 (45), an upstream target of the inflammatory signaling pathway enriched in subtypes A and B. The effects of MTX therapy on molecular signatures were largely restricted to proinflammatory pathways and immune cell subsets. In addition, MTX has recently been discovered to inhibit the JAK/STAT pathway, while many of its side effects are likely to be related to the folate pathway (46). The IL-23 receptor relies on a heterodimer of Janus kinase 2 (JAK2) and tyrosine kinase 2 (TYK2) for signal transduction, thereby highlighting the role of JAKs in the therapeutical potential of JAK inhibitors (47, 48). Moreover, Ishizaki et al. observed that TYK2-deficient mice injected with IL-23 showed significantly reduced ear erythema and epidermal hyperplasia compared to wild-type mice (49). A lack of TYK2 also impaired the infiltration of various immune cells into the skin and the production of IL-17 and IL-22. The JAK/STAT and IL-12/23 pathways have similar contributions to the progression of psoriasis. Thus, it ought to be prudent to research whether the loss of efficacy or delayed treatment resistance can be attributed to the conversion of skin molecular patterns.

Nevertheless, the weak response to TNF-α and IL-17RA inhibitors in patients with subtypes A compared with that for subtype C seemed to contradict their enrichment of numerous inflammatory pathways and underlying pathophysiology. This might be attributed to a paradoxical reaction, in which the disease worsens during treatment with targeted biological drugs. Two main hypotheses have been proposed for this contradictory response (50). One theory is the involvement of TNF-α and IFN-α cross-regulation. Palucka et al. demonstrated that blocking endogenous TNF-α results in increased IFN-γ production by pDC and subsequent T-cell activation resulting in increased TNF-α production (51). The skin lesions in patients with psoriasis induced by TNF-α inhibitors are characterized by IFN-α overexpression compared with those in patients with psoriasis vulgaris. Another hypothesis posits that following TNF-α inhibition, Th17 cells are enhanced and regulatory T cells are downregulated, leading to increased production of the Th17 cytokine IL-22 (52). Both pathways produce cytokines that act on keratinocytes and generate a proinflammatory cycle, leading to suboptimal treatment of psoriasis. This suggests that although targeted therapy for specific cytokines has achieved good efficacy, some people may experience negative feedback after long-term targeted therapy, leading to the loss of treatment effectiveness. Moreover, inhibiting single downstream cytokines (TNF-α and IL-17) to prevent inflammatory response and reverse the disease outcome entirely is challenging. Thus, active intervention in the dysregulation of upstream targets (IL-12/23) is warranted.

Our study has some limitations. First, this study was conducted in different clinical environments from different public datasets; more meta-data is required, but this may prove challenging. Second, owing to the lack of complete annotation for each psoriasis sample, we could not address the association of each psoriasis subtype with clinical factors, such as autoantibody levels. Finally, these differences across responders and non-responders of subtypes were not statistically significant owing to the limited sample size of the clinical response in patients with psoriasis.

## 5 Conclusions

In summary, we deconvoluted psoriasis skin tissues into pathologically discrete subsets by combining skin transcriptomic data with machine learning algorithms. We described their different molecular and cellular characteristics regarding treatment response. Our results provide critical insights into distinct and shared mechanistic features of psoriasis to determine the pathobiological approaches for benefiting drug-resistant patients.

## Data availability statement

The original contributions presented in the study are included in the article/**Supplementary Material**. Further inquiries can be directed to the corresponding author.

## Ethics statement

This study used publicly available data from the Gene Expression Omnibus (GEO) database. All subjects in the original studies have provided informed consent, so further ethical approval is not required.

## Author contributions

SZ: Writing – original draft. MC: Writing – original draft. LZhe: Writing – original draft. CanW: Writing – review & editing. RZ: Writing – review & editing. SS: Writing – review & editing. JH: Writing – review & editing. LZha: Writing – review & editing. CaiW: Writing – review & editing. XL: Writing – review & editing.

## Supplementary material

Supplementary Image 1 Principal component analysis (PCA) plots with or without the elimination of batch effects. **(A)** PCA before batch effect adjustment for the training microarray datasets. Samples from the different datasets cluster together. **(B)** PCA after batch effect adjustment for the training microarray datasets. Samples from different datasets overlap.
