# Supplementary figures and images for: Deep analysis of skin molecular heterogeneities and their significance on the precise treatment of patients with psoriasis
Source: Front Immunol. 2024 Mar 1;15:1326502. doi: 10.3389/fimmu.2024.1326502 (PMC10940483; doi:10.3389/fimmu.2024.1326502)

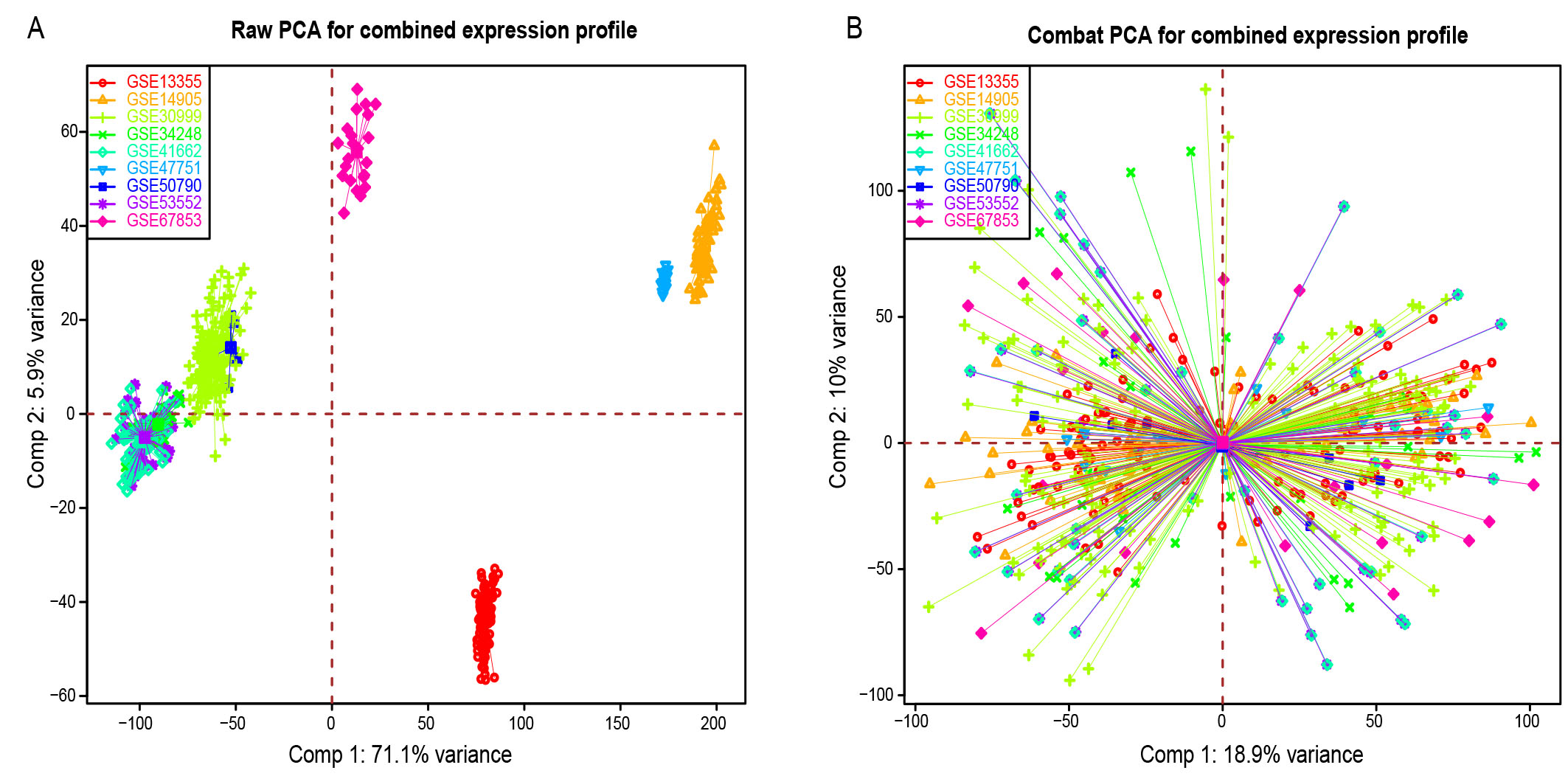

Supplement: Supplementary Image 1 — Principal component analysis (PCA) plots with or without the elimination of batch effects. (A) PCA before batch effect adjustment for the training microarray datasets. Samples from the different datasets cluster together. (B) PCA after batch effect adjustment for the training microarray datasets. Samples from different datasets overlap. [file Image_1.jpeg]
