# Supplementary material for: Deep analysis of skin molecular heterogeneities and their significance on the precise treatment of patients with psoriasis
Source: Front Immunol. 2024 Mar 1;15:1326502. doi: 10.3389/fimmu.2024.1326502 (PMC10940483; doi:10.3389/fimmu.2024.1326502)
Supplement: Supplementary file 2 [file Table_1.docx]

**Supplementary Table 1. Psoriasis sample info.**

| **Data set** | **Subjects** | **Experiment type** | **Platforms** | **Collection site** | **Reference** |  |  |  |  |  |  |  |  |
| --- | --- | --- | --- | --- | --- | --- | --- | --- | --- | --- | --- | --- | --- |
| **microarray** | 250 LS vs 250 NL |  |  |  |  |  |  |  |  |  |  |  |  |
| Accession: GSE30999 | 81 LS vs 81 NL | Expression profiling by array | GPL570 | Janssen R&D, USA | Suárez-Fariñas et al. | PMID: 22763790 | [https://www.ncbi.nlm.nih.gov/geo/query/acc.cgi?acc=GSE30999](https://www.ncbi.nlm.nih.gov/geo/query/acc.cgi?acc=GSE30999) |  |  |  |  |  |  |
| Accession: GSE13355 | 58 LS vs 58 NL | Expression profiling by array | GPL570 | University of Michigan, USA | Nair et al. | PMID: 19169254 | <https://www.ncbi.nlm.nih.gov/geo/query/acc.cgi?acc=GSE13355> |  |  |  |  |  |  |
| Accession: GSE14905 | 28 LS vs28 NL | Expression profiling by array | GPL570 | MedImmune, LLC, USA | Yao et al. | PMID: 18648529 | [https://www.ncbi.nlm.nih.gov/geo/query/acc.cgi?acc=GSE14905](https://www.ncbi.nlm.nih.gov/geo/query/acc.cgi?acc=GSE14905) |  |  |  |  |  |  |
| Accession: GSE41662 | 24 LS vs 24 NL | Expression profiling by array | GPL570 | BJ Group, LLC, USA | Bigler et al. | PMID: 23308107 | [https://www.ncbi.nlm.nih.gov/geo/query/acc.cgi?acc=GSE41662](https://www.ncbi.nlm.nih.gov/geo/query/acc.cgi?acc=GSE41662) |  |  |  |  |  |  |
| Accession: GSE53552 | 24 LS vs 24 NL | Expression profiling by array | GPL570 | BJ Group, LLC, USA | Bigler et al. | PMID: 24646743 | [https://www.ncbi.nlm.nih.gov/geo/query/acc.cgi?acc=GSE53552](https://www.ncbi.nlm.nih.gov/geo/query/acc.cgi?acc=GSE53552) |  |  |  |  |  |  |
| Accession: GSE34248 | 14 LS vs 14 NL | Expression profiling by array | GPL570 | BJ Group, LLC, USA | Bigler et al. | PMID: 23308107 | [https://www.ncbi.nlm.nih.gov/geo/query/acc.cgi?acc=GSE34248](https://www.ncbi.nlm.nih.gov/geo/query/acc.cgi?acc=GSE34248) |  |  |  |  |  |  |
| Accession: GSE67853 | 11 LS vs 11 NL | Expression profiling by array | GPL570 | Rockefeller University, USA | Kim et al. | PMID: 26763436 | [https://www.ncbi.nlm.nih.gov/geo/query/acc.cgi?acc=GSE67853](https://www.ncbi.nlm.nih.gov/geo/query/acc.cgi?acc=GSE67853) |  |  |  |  |  |  |
| Accession: GSE47751 | 6 LS vs 6 NL | Expression profiling by array | GPL570 | Harvard University, USA | Johnston et al. | PMID: 24601997 | [https://www.ncbi.nlm.nih.gov/geo/query/acc.cgi?acc=GSE47751](https://www.ncbi.nlm.nih.gov/geo/query/acc.cgi?acc=GSE47751) |  |  |  |  |  |  |
| Accession: GSE50790 | 4 LS vs 4 NL | Expression profiling by array | GPL570 | Harvard University, USA | Swindell et al. | PMID: 22479649 | [https://www.ncbi.nlm.nih.gov/geo/query/acc.cgi?acc=GSE50790](https://www.ncbi.nlm.nih.gov/geo/query/acc.cgi?acc=GSE50790) |  |  |  |  |  |  |
| **Treatment set (microarray)** |  |  |  |  |  |  |  |  |  |  |  |  |  |
| Accession: GSE117468 | Ustekinumab  (14 Response vs 1 Non-response)  Brodalumab  (61 Response vs 12 Non-response) | Expression profiling by array | GPL570 | Mount SinaiSchool of Medicine, USA | Tomalin et al. | PMID: 31883845 | <https://www.ncbi.nlm.nih.gov/geo/query/acc.cgi?acc=GSE117468> |  |  |  |  |  |  |
|  | Brodalumab |  |  |  |  |  |  |  |  |  |  |  |  |
| Accession: GSE117239 | Ustekinumab  (31 Response vs 11 Non-response)  Etanercept  (20 Response vs 9 Non-response) | Expression profiling by array | GPL570 | Rockefeller University, USA | Brodmerkel et al. | PMID: 30703387 | <https://www.ncbi.nlm.nih.gov/geo/query/acc.cgi?acc=GSE117239> |  |  |  |  |  |  |
|  |  |  |  |  |  |  |  |  |  |  |  |  |  |
| Accession: GSE106992 | Ustekinumab  (19 Response vs 9 Non-response)  Etanercept  (19 Response vs 10 Non-response) | Expression profiling by array | GPL570 | Rockefeller University, USA | Brodmerkel et al. | PMID: 30703387 | <https://www.ncbi.nlm.nih.gov/geo/query/acc.cgi?acc=GSE106992> |  |  |  |  |  |  |
| Accession: GSE41663 | Etanercept  (6 Response vs 2 Non-response) | Expression profiling by array | GPL570 | BJ Group, LLC, USA | Bigler et al. | PMID: 23308107 | <https://www.ncbi.nlm.nih.gov/geo/query/acc.cgi?acc=GSE41663> |  |  |  |  |  |  |
| Accession: GSE85034 | Methotrexate  (4 Response vs 11 Non-response) | Expression profiling by array | GPL10558 | Rockefeller University, USA | Correa da Rosa et al. | PMID: 27354683 | <https://www.ncbi.nlm.nih.gov/geo/query/acc.cgi?acc=GSE85034> |  |  |  |  |  |  |
